# Institutional mortality rate and cause of death at health facilities in Ghana between 2014 and 2018

**DOI:** 10.1371/journal.pone.0256515

**Published:** 2021-09-08

**Authors:** Adobea Yaa Owusu, Sandra Boatemaa Kushitor, Anthony Adofo Ofosu, Mawuli Komla Kushitor, Atsu Ayi, John Koku Awoonor-Williams

**Affiliations:** 1 Institute of Statistical, Social and Economic Research (ISSER), College of Humanities, University of Ghana, Legon, Ghana; 2 Food Security Initiative, Stellenbosch University, Stellenbosch, South Africa; 3 Ghana Health Service, Accra, Ghana; 4 Department of Health, Policy Planning, and Management, School of Public Health, University of Health and Allied Sciences, Hohoe, Ghana; 5 Ghana Health Service, Accra, Ghana; 6 Policy Planning Monitoring and Evaluation Division, Ghana Health Service, PMB, Accra, Ghana; National Cancer Center, Japan, JAPAN

## Abstract

**Background:**

The epidemiological transition, touted as occurring in Ghana, requires research that tracks the changing patterns of diseases in order to capture the trend and improve healthcare delivery. This study examines national trends in mortality rate and cause of death at health facilities in Ghana between 2014 and 2018.

**Methods:**

Institutional mortality data and cause of death from 2014–2018 were sourced from the Ghana Health Service’s District Health Information Management System. The latter collates healthcare service data routinely from government and non-governmental health institutions in Ghana yearly. The institutional mortality rate was estimated using guidelines from the Ghana Health Service. Percent change in mortality was examined for 2014 and 2018. In addition, cause of death data were available for 2017 and 2018. The World Health Organisation’s 11^th^ International Classification for Diseases (ICD-11) was used to group the cause of death.

**Results:**

Institutional mortality decreased by 7% nationally over the study period. However, four out of ten regions (Greater Accra, Volta, Upper East, and Upper West) recorded increases in institutional mortality. The Upper East (17%) and Volta regions (13%) recorded the highest increase. Chronic non-communicable diseases (NCDs) were the leading cause of death in 2017 (25%) and 2018 (20%). This was followed by certain infectious and parasitic diseases (15% for both years) and respiratory infections (10% in 2017 and 13% in 2018). Among the NCDs, hypertension was the leading cause of death with 2,243 and 2,472 cases in 2017 and 2018. Other (non-ischemic) heart diseases and diabetes were the second and third leading NCDs. Septicaemia, tuberculosis and pneumonia were the predominant infectious diseases. Regional variations existed in the cause of death. NCDs showed more urban-region bias while infectious diseases presented more rural-region bias.

**Conclusions:**

This study examined national trends in mortality rate and cause of death at health facilities in Ghana. Ghana recorded a decrease in institutional mortality throughout the study. NCDs and infections were the leading causes of death, giving a double-burden of diseases. There is a need to enhance efforts towards healthcare and health promotion programmes for NCDs and infectious diseases at facility and community levels as outlined in the 2020 National Health Policy of Ghana.

## Introduction

Sub-Saharan Africa is experiencing an epidemiological transition [1]. This transition is characterised by a high burden of both infectious and chronic non-communicable diseases, which has resulted in a disproportionate burden of morbidity and mortality. The region had only 14% of the world’s population as of 2019 [2], yet 25% of the world’s disease burden [3, 4]. Despite the increase in the burden of disease, countries in the region are unable to monitor and provide knowledge of the patterns and trends of morbidity and mortality due to the lack of reliable and accessible data systems. This can thwart the efforts towards achieving the sustainable development goals.

Although Ghana is still struggling with communicable diseases, researchers note a significant shift in the pattern to non-communicable diseases, with cardiovascular diseases like stroke and hypertension at the forefront [5, 6]. This situation has been described as the double burden of diseases [7]. In 2014, the major causes of death included maternal mortality, cancer, stroke, heart diseases and related complications, HIV/AIDS, malaria, tuberculosis, diabetes, cholera, and road traffic accidents [8]. Unfortunately, most mortality analyses in Ghana are cross-sectional and localised geographically [5]. This limits their suitability to the national response to healthcare. This study used mortality data from health facilities for a five-year period and provides a longitudinal perspective of disease patterns in the country.

Ghana’s healthcare system is not robust enough to fully meet the healthcare needs/demands of the Ghanaian populace [9, 10]. From personnel to logistics and institutions, the nation is lagging, contributing to some preventable deaths [11]. Higher mortality is recorded in rural areas. More deaths are recorded from referral cases; mostly from less endowed healthcare facilities in rural areas to urban ones. Lee et al. [12] observed that almost half (43.4%) of referrals from other hospitals contributed to the maternal deaths at Komfo Anokye Teaching Hospital, Ghana’s second tertiary hospital and referral centre. Furthermore, poor transportation systems and challenges in community health clinics such as poor emergency response and low skilled health personnel, are some major healthcare delivery challenges in rural Ghana [13].

Moreover, Agyemang et al.’s [5] study at the same facility showed that about 45% of stroke patients admitted to the hospital died due to failure-to-rescue related challenges [14]. This study examined institutional mortality rates from 2014 to 2018 and cause of death for 2017 to 2018 using data from the Ghana Health Service. The research builds on previous studies that have examined either infectious diseases or NCDs’ patterns in Ghana. Most importantly, this study fills the gap by providing knowledge of the burden of both infectious and non-communicable diseases in Ghana. The findings are important for health policy focus, planning, monitoring and evaluation and can support the 2020 National Health Policy.

## Methods

### Data source

The data for this study were retrieved from the Policy, Planning, Monitoring and Evaluation Division (PPMED) of the Ghana Health Service. They were derived from the Ghana Health Service’s District Health Information Management System (DHIMS2), a web-based system adapted from the dhis2 software [15]. The DHIMS2 was started in 2012 and is being used to collect and analyse routine health service data. Health workers in government facilities, as well as most private and Christian Health Association Ghana (CHAG) facilities in districts, are expected to provide information on a daily basis on deaths and their cause. The data are automatically aggregated by districts and regions. In January 2019, Ghana was divided into 16 regions, from 10 regions. The data for this study, which were already collected based on the ten regions as of the close of 2018, were unpacked to fit the 16 regions by the Ghana Health Service. However, we presented the data by the 10 regions by combining the new regions to their former regions to provide clarity and better understanding of the issues, disparities and context. We requested the Ghana Health Service to unpack the data to their original ten regions in order that we would be unbiased. We did the regrouping of the data to their original regions for the data collection because we expect that if we used the new regions, the findings from our analysis in terms of regional positions would be different from what prevailed if the old regions were used.

To a large extent, the regional division did not take account of health facilities or human resource challenges, which has resulted in huge gaps in health provision. For instance, in the North East Region, which originally belonged to the parent Northern Region together with the newly created Savannah Region, after the separation, there were virtually no health facilities left there except a few Community-based Health Planning and Services (CHPS) compounds and a CHAG facility. We focus on 2017 and 2018 for the cause of death because a review of the data indicates that the DHIMS2 process became more stabilised over those two years, compared to the initial three years (2014–2016).

### Data analysis

Each year, the institutional mortality rate was estimated using the Ghana Health Service Standard Operating Procedures (SOP) for Health Information. Institutional mortality rate was estimated by dividing all institutional deaths by all institutional admissions per 1000 [(all institutional deaths/all institutional admission)*1000]. The health workers provide the underlying (main) cause of death when entering data into the DHIMS2. One hundred and twenty-four diseases were listed. The World Health Organisation’s (WHO) [16] ICD-11 for Mortality and Morbidity statistics, was used to group the causes of death (Table 1). However, some diseases were grouped based on the current increase in prevalence, the long history of the condition in Ghana, and the limited attention to these conditions. For a long history, malaria infections were grouped as a unique category instead of adding them to certain infectious and parasitic diseases [17]. Hypertension, diabetes, and heart diseases were grouped as chronic non-communicable diseases (NCDs) due to their rising prevalence in the country [9, 18]. Disease-specific death rates at health facilities per region were calculated using the total admissions as the denominator. For example, the malaria mortality rate for the Eastern Region was calculated as [(Malaria deaths in the Eastern Region/All hospital admissions in the Eastern Region) *1000).

**Table 1:** Disease groupings for the cause of death analysis using the World Health Organisation’s ICD-11 for mortality and morbidity statistics.

Disease grouping	Underlying cause of death
**Alcohol-related disorder**	Alcohol poisoning, alcohol use disorders
**Cancers/neoplasms**	Benign neoplasms, diseases of the eye and adnexa, Leukaemia, malignant melanoma of the skin, malignant neoplasm of bladder, malignant neoplasm of breast, malignant neoplasm of colon, rectum and anus, malignant neoplasm of the larynx, malignant neoplasm of lip, oral cavity and pharynx, malignant neoplasm of liver and intrahepatic bile ducts, malignant neoplasm of meninges, brain and other parts of central nervous system, malignant neoplasm of oesophagus, malignant neoplasm of other and unspecified parts of uterus, malignant neoplasm of ovary, malignant neoplasm of pancreas, malignant neoplasm of prostate, malignant neoplasm of stomach, malignant neoplasm of trachea, bronchus and lung, multiple myeloma and malignant plasma cell neoplasms, Non-Hodgkin lymphoma, other and unspecified malignant neoplasms
**Certain infections and parasites**	Hepatitis B, meningococcal infection, other tuberculosis, other viral haemorrhagic fevers, tetanus, tuberculosis, typhoid and paratyphoid, unspecified viral hepatitis, yellow fever, septicaemia
**Chronic non-communicable diseases**	Hypertension, diabetes, Ischaemic heart diseases, Other heart diseases
**Circulatory system**	Acute rheumatic fever and chronic rheumatic heart diseases
	Cerebrovascular diseases
	Other and unspecified diseases of the circulatory system
**Diseases of the ear and mastoid**	Diseases of the ear and mastoid process
**Diseases of the digestive system**	Appendicitis, cholera, diseases of the musculoskeletal system and connective tissue, gastric and duodenal ulcer, liver cirrhosis, other and unspecified diarrhoeal diseases
**Diseases of the nervous system**	Other diseases of the nervous system
**Diseases of the genitourinary system**	Glomerular and renal tubulo-interstitial diseases, malignant neoplasm of cervix uteri, other and unspecified diseases of the genitourinary system
**Diseases of the skin**	Diseases of the skin and subcutaneous tissue
**Diseases of the visual system**	Diseases of the eye and adnexa
**External causes of morbidity**	Accidental drowning and submersion, accidental poisoning by and exposure to noxious substances, assault, conflict and war, drug poisoning, exposure to forces of nature, exposure to smoke, fire and flames, falls, food poisoning, intentional self-harm, other and unspecified poisoning
**Foetal deaths**	Certain conditions originating in the perinatal period: Birth trauma, prematurity, sepsis and other infectious conditions of the new-born
	Foetus and new-born affected by maternal factors and by complications of pregnancy, labour and delivery: Intrauterine hypoxia and birth asphyxia, Other and unspecified perinatal conditions, other direct obstetric deaths
	Congenital malformations, deformations and chromosomal abnormalities: Congenital hydrocephalus and spine bifida, congenital malformations of the heart down syndrome and other chromosomal abnormalities, other and unspecified congenital malformations
	Disorders relating to the length of gestation and foetal growth: Low birth weight, other and unspecified disorders relating to the length of gestation and foetal growth
**HIV**	Human immunodeficiency virus [HIV] disease
	HIV disease with tuberculosis
**Malaria**	Malaria, parasitologically confirmed, other and unspecified malaria
**Malnutrition**	Protein-energy malnutrition, anaemias, other and unspecified endocrine, nutritional and metabolic diseases
**Mental, behavioural and neurodevelopmental disorders**	Drug use disorders, other mental and behavioural disorders
**Neglected tropical diseases (NTDs)**	Meningitis
**Pregnancy, childbirth and puerperium**	Indirect obstetric deaths, maternal haemorrhage, maternal hypertensive disorders, maternal sepsis, obstructed labour, pregnancy with abortive outcome
**Respiratory infections**	Chronic lower respiratory diseases, influenza, other and unspecified diseases of the respiratory system, other and unspecified infectious diseases, pneumonia
**Road accidents**	Road traffic accidents, other transport accidents
**Sexually transmitted Infections (STIs)**	Syphilis, other and unspecified infections with a predominantly sexual mode of transmission
**Symptoms, signs and abnormal clinical and laboratory findings, not elsewhere classified**	Symptoms, signs and abnormal clinical and laboratory findings, not elsewhere classified
**Unspecified**	Not applicable

### Ethics

Consistent with earlier work [19–21], studies using anonymous data are exempt from ethical review.

## Results

### Trends in institutional mortality rate

**Fig 1:**
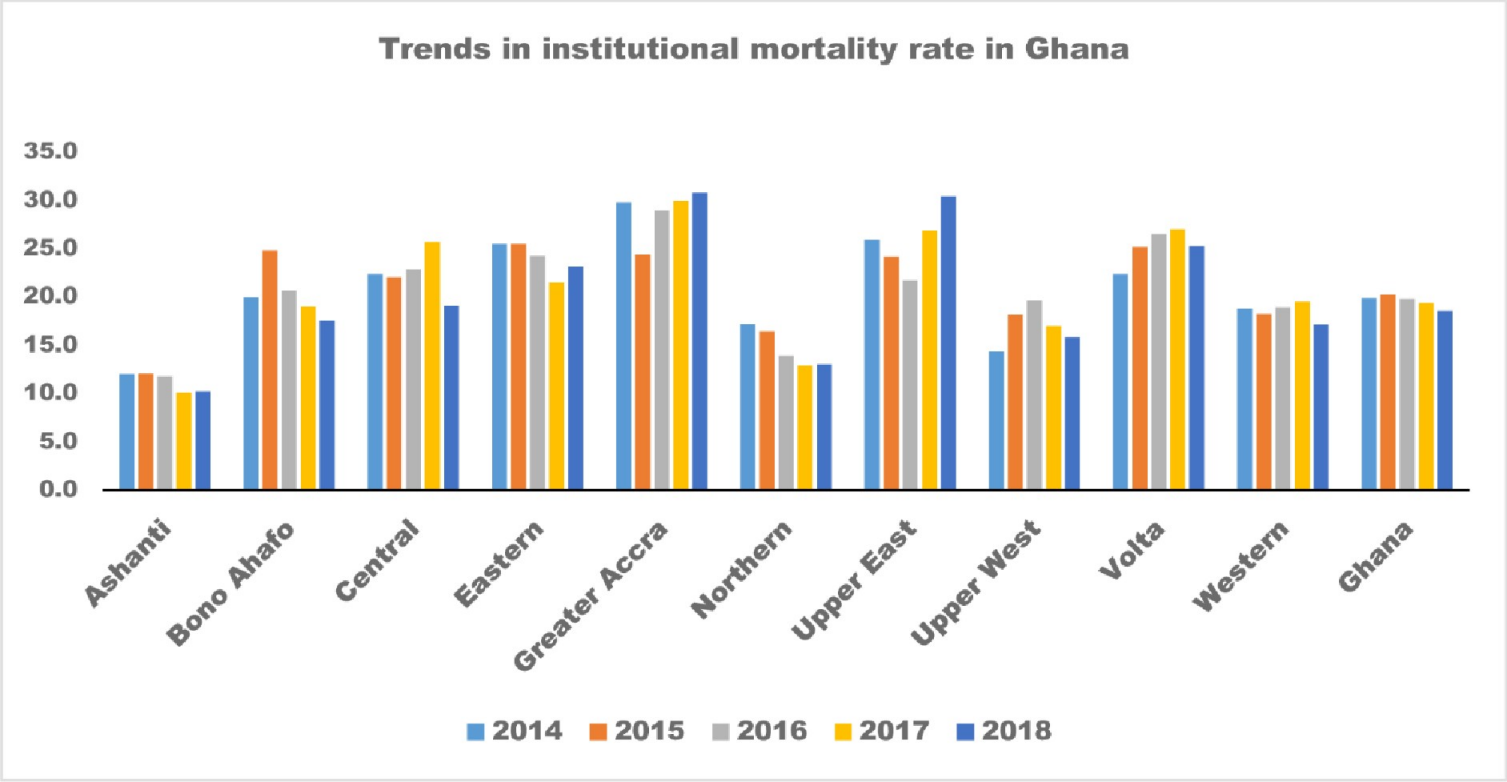
Trends in Institutional mortality rate in Ghana, 2014 and 2018, by regions. Source: Computed by authors based on Ghana Health Service’s DHIMS2, 2020.

Between 2014 and 2018, institutional mortality at the national level was about 20 deaths per 1000 admissions (Fig 1). However, variations by regions were noticed (Fig 1, Table 2). The highest reductions were recorded in the Northern Region, followed by the Ashanti and Central Regions. Nevertheless, the Greater Accra, Volta, Upper East and Upper West regions recorded an increase in institutional mortality during the five years (Fig 1, Table 2).

**Table 2:** Percent change in institutional mortality rate in Ghana between 2014 and 2018, by region.

Index	Institutional mortality		
Year	2014	2018	% Change
**Ashanti**	12.0	10.2	-15.0
**Brong Ahafo**	19.9	17.5	-12.1
**Central**	22.3	19.1	-14.4
**Eastern**	25.5	23.1	-9.4
**Greater Accra**	29.7	30.8	3.7
**Northern**	17.2	13.0	-24.4
**Upper East**	25.9	30.4	17.4
**Upper West**	14.4	15.8	9.7
**Volta**	22.3	25.2	13.0
**Western**	18.8	17.1	-9.0
**Ghana**	**19.9**	**18.5**	**-7.0**

Source: Computed by authors based on Ghana Health Service’s DHIMS2, 2020.

### Cause of death at health facilities at the national level in 2017 and 2018

The health facilities in the country reported a total of 14,860 for 2017 and 24,714 for 2018 cause-specific deaths. The 25 disease groups are ranked by the mortality rate (Table 3). The leading cause of death nationally for both years was chronic non-communicable diseases, with 3,754 in 2017 (3.0 deaths per 1000 admissions) and 4,960 cases in 2018 (2.5 deaths per 1000 admissions). Certain infections and parasites, the second leading cause of death nationally for both years, recorded a mortality rate of 2.3 deaths per 1000 admissions in 2017. The third leading cause of death nationally for both years, was respiratory infections. The mortality rate of respiratory infections was 2 per 1000 admissions in 2017. The first nine leading causes of death were the same for both years. HIV was among the ten leading causes of death in 2017 but placed 12^th^ in 2018 (Table 3).

**Table 3:** Ranked mortality rate by cause of death at health facilities Ghana, 2017 and 2018.

2017		2018	
Disease groups	Mortality rate	Disease groups	Mortality rate
**Chronic non-communicable diseases**	3.0	**Chronic non-communicable diseases**	2.5
**Certain infections and parasites**	2.3	**Certain infections and parasites**	1.4
**Respiratory infections**	2.0	**Respiratory infections**	1.0
**Digestive system**	1.3	**Digestive system**	0.8
**Diseases of the circulatory system**	1.3	**Diseases of the circulatory system**	0.6
**Malnutrition**	1.1	**Malnutrition**	0.6
**Foetal deaths**	0.9	**Foetal deaths**	0.5
**Cancers**	0.7	**Cancers**	0.4
**Diseases of the genitourinary system**	0.5	**Diseases of the genitourinary system**	0.3
**Symptoms, signs and abnormal clinical and laboratory findings, not elsewhere classified**	0.4	**HIV**	0.3
**External falls**	0.4	**Symptoms, signs and abnormal clinical and laboratory findings, not elsewhere classified**	0.3
**HIV**	0.3	**Malaria**	0.2
**Malaria**	0.3	**External falls**	0.2
**Diseases of the nervous system**	0.2	**Alcohol**	0.1
**Diseases of the skin**	0.1	**Diseases of the nervous system**	0.1
**Alcohol**	0.1	**Diseases of the skin**	0.1
**Road accidents**	0.1	**Neglected tropical diseases**	0.1
**Neglected tropical diseases**	0.1	**Road accidents**	0.1
**Pregnancy, childbirth and puerperium**	0.1	**Pregnancy, childbirth and puerperium**	0.0
**Diseases of the musculoskeletal system and connective tissue**	0.0	**Mental, behavioural and neurodevelopmental disorders**	0.0
**Mental, behavioural and neurodevelopmental disorders**	0.0	**Diseases of the musculoskeletal system and connective tissue**	0.0
**Diseases of the ear and mastoid process**	0.0	**Unspecified**	0.0
**Diseases of the visual system**	0.0	**Diseases of the ear and mastoid process**	0.0
**Sexually Transmitted Infections**	0.0	**Sexually Transmitted Infections**	0.0
**Unspecified**	0.0	**Diseases of the visual system**	0.0

Source: Computed by authors based on Ghana Health Service’s DHIMS2, 2020

### Cause of death at health facilities at the regional level 2017 and 2018

In both years, hypertension was the leading NCD cause of death (Tables 4 and 5). Hypertension deaths were very high in the Greater Accra (4.8 deaths per 1000 admissions), Eastern (2.5 deaths per 1000 admissions), Western (1.7 deaths per 1000 admissions), Brong Ahafo (1.6 deaths per 1000 admissions) regions compared to the other regions in 2018 (S1 Table). Pneumonia was the predominant respiratory infection in both 2018 and 2017. The Upper East (2.6 deaths per 1000 admissions) region recorded more deaths from pneumonia than the other regions in 2018 (S1 Table). In 2017, pneumonia deaths were highest in the Volta Region (4.6 deaths per 1000 admissions), and lowest in the Ashanti (0.2 deaths per 1000 admissions), and Northern (0.3 deaths per 1000 admissions) regions (S2 Table). Other and unspecified respiratory diseases were the second highest cause of deaths among respiratory infections in 2017 and 2018 (Tables 4 and 5).

**Table 4:** Mortality rate by disease groups in 2018 at health facilities in Ghana, by region.

Disease group	Ashanti	Brong Ahafo	Central	Eastern	Greater Accra	Northern	Upper East	Upper West	Volta	Western
**Alcohol related disorder**	0.1	0.2	0.1	0.2	0.2	0.0	0.4	0.0	0.0	0.1
**Cancers/neoplasms**	0.2	0.7	0.7	0.8	2.9	0.1	0.4	0.4	0.1	0.4
**Certain infections and parasites**	1.3	3.4	2.2	3.3	3.4	0.9	3.5	1.8	0.3	1.9
**Chronic non communicable diseases**	1.6	3.5	2.9	5.0	8.6	0.7	3.1	1.0	0.3	2.6
**Circulatory system**	0.4	1.3	1.2	2.7	3.0	0.3	1.4	0.5	0.2	0.6
**Diseases of the digestive system**	0.6	1.9	1.0	1.5	2.0	0.5	3.1	1.4	0.2	0.9
**Diseases of the ear and mastoid process**	0.0	0.0	0.0	0.0	0.0	0.0	0.0	0.0	0.0	0.0
**Diseases of the genitourinary system**	0.2	0.6	0.6	0.7	1.5	0.1	0.6	0.4	0.0	0.2
**Diseases of the musculoskeletal system and connective tissue**	0.0	0.0	0.0	0.0	0.1	0.0	0.1	0.0	0.0	0.0
**Diseases of the nervous system**	0.0	0.2	0.2	0.2	0.5	0.0	0.1	0.1	0.0	0.2
**Diseases of the skin**	0.0	0.1	0.3	0.1	0.3	0.0	0.3	0.1	0.0	0.1
**Diseases of the visual system**	0.0	0.0	0.0	0.0	0.0	0.0	0.0	0.0	0.0	0.0
**External causes of morbidity**	0.1	0.6	0.2	0.3	0.4	0.3	1.0	0.6	0.0	0.3
**Fetal deaths**	0.3	1.1	0.4	0.9	2.4	0.5	2.2	0.9	0.1	0.7
**HIV**	0.3	0.7	0.3	0.4	0.5	0.0	0.4	0.4	0.0	0.2
**Malaria**	0.1	0.4	0.2	0.2	0.1	0.7	0.8	0.2	0.0	0.2
**Malnutrition**	0.5	1.2	1.0	1.2	1.2	0.9	2.3	0.7	0.1	1.4
**Mental, behavioral and neurodevelopmental disorders**	0.0	0.0	0.0	0.0	0.1	0.0	0.0	0.0	0.0	0.0
**Neglected tropical diseases**	0.0	0.2	0.1	0.2	0.1	0.1	0.3	0.2	0.0	0.0
**Pregnancy, childbirth and puerperium**	0.0	0.1	0.1	0.1	0.0	0.0	0.0	0.0	0.0	0.1
**Respiratory infections**	1.0	2.9	1.9	2.4	3.5	1.0	3.6	1.2	0.3	1.4
**Road accidents**	0.0	0.1	0.1	0.1	0.1	0.0	0.9	0.1	0.0	0.1
**Sexually transmitted infections**	0.0	0.0	0.0	0.0	0.0	0.0	0.0	0.0	0.0	0.0
**Symptoms, signs and abnormal clinical and laboratory findings, not elsewhere classified**	0.1	0.5	0.4	0.4	0.7	0.2	0.9	0.3	0.1	0.3
**Unspecified**	0.0	0.0	0.0	0.0	0.0	0.0	0.0	0.0	0.0	0.0

**Table 5:** Mortality rate by disease groups in 2017 at health facilities in Ghana, by region.

Disease groups	Ashanti	Brong Ahafo	Central	Eastern	Greater Accra	Northern	Upper East	Upper West	Volta	Western
**Alcohol related disorder**	0.1	0.1	0.1	0.1	0.6	0.0	0.1	0.1	0.2	0.1
**Cancers/neoplasms**	0.1	0.4	0.6	0.3	2.4	0.1	0.1	0.5	0.3	0.3
**Certain infections and parasites**	0.4	1.7	1.6	1.8	5.1	0.3	0.7	1.5	1.2	1.5
**Chronic non communicable diseases**	0.5	1.9	2.1	1.9	13.6	0.2	0.7	1.0	2.5	2.7
**Circulatory system**	0.1	0.5	0.7	0.9	3.2	0.1	0.1	0.3	0.8	0.4
**Diseases of the digestive system**	0.2	0.8	0.9	0.6	2.9	0.3	0.5	0.7	0.8	0.7
**Diseases of the ear and mastoid process**	0.0	0.0	0.0	0.0	0.0	0.0	0.0	0.0	0.0	0.0
**Diseases of the genitourinary system**	0.1	0.3	0.5	0.2	1.6	0.0	0.1	0.3	0.3	0.3
**Diseases of the musculoskeletal system and connective tissue**	0.0	0.0	0.0	0.0	0.1	0.0	0.0	0.0	0.0	0.0
**Diseases of the nervous system**	0.0	0.2	0.1	0.1	0.5	0.0	0.0	0.1	0.1	0.2
**Diseases of the skin**	0.0	0.1	0.1	0.1	0.5	0.0	0.0	0.1	0.1	0.1
**Diseases of the visual system**	0.0	0.0	0.0	0.0	0.0	0.0	0.0	0.0	0.0	0.0
**External falls**	0.0	0.2	0.2	0.2	0.4	0.1	0.2	0.4	0.3	0.2
**Foetal deaths**	0.1	0.7	0.3	0.5	2.3	0.1	0.3	0.8	0.2	0.3
**HIV**	0.1	0.3	0.2	0.4	1.1	0.0	0.1	0.4	0.1	0.1
**Malaria**	0.0	0.2	0.3	0.2	0.4	0.4	0.2	0.3	0.2	0.2
**Malnutrition**	0.2	0.9	0.7	0.6	1.8	0.4	0.6	0.5	0.5	0.9
**Mental, behavioral and neurodevelopmental disorders**	0.0	0.0	0.0	0.0	0.2	0.0	0.0	0.0	0.0	0.0
**Neglected tropical diseases**	0.0	0.1	0.1	0.1	0.2	0.1	0.0	0.2	0.1	0.1
**Pregnancy, childbirth and puerperium**	0.0	0.1	0.1	0.0	0.2	0.0	0.0	0.0	0.0	0.1
**Respiratory infections**	0.3	1.2	1.2	1.2	3.1	0.3	0.6	0.9	1.1	0.9
**Road accidents**	0.0	0.1	0.0	0.1	0.0	0.0	0.0	0.2	0.1	0.1
**Sexually transmitted infections**	0.0	0.0	0.0	0.0	0.0	0.0	0.0	0.0	0.0	0.0
**Symptoms, signs and abnormal clinical and laboratory findings, not elsewhere classified**	0.1	0.2	0.1	0.2	1.4	0.1	0.0	0.2	0.3	0.3
**Unspecified**	0.1	0.0	0.0	0.0	0.0	0.0	0.0	0.0	0.0	0.0

Cerebrovascular diseases were the leading circulatory diseases causing death in 2018, with 1,893 deaths. These diseases affect blood vessels and blood supply to the brains. Diseases of blood-forming organs, the fourth cause of death, was measured with septicaemia, a blood poisoning condition caused by bacteria. The regions with septicaemia death rate above 1.5 in 2018 were the Upper East (2.1 deaths per 1000 admissions), Greater Accra (1.7 deaths per 1000 admissions), and Brong Ahafo (1.6 deaths per 1000 admissions) (Table 4).

Prematurity, intrauterine hypoxia and birth asphyxia, and other perinatal conditions were the underlying conditions that caused the most foetal death for both years. In 2018, sepsis and other new-born infection were also among the leading causes of foetal deaths (Table 4). The Greater Accra, Northern, Upper East, and Brong Ahafo Regions, compared to the others, recorded more deaths from these conditions. In 2018, for example, the Upper East Region had a mortality rate of 0.4 deaths per 1000 admissions from prematurity, 1.0 deaths per 1000 admissions from intrauterine hypoxia and birth asphyxia, and 0.6 deaths per 1000 admissions) from sepsis and other infections (S1 Table). The main malnutrition conditions were anaemia and protein-energy malnutrition. Anaemia recorded more deaths in both years than protein-energy malnutrition. Regions with anaemia mortality rate above 1 in 2018 were Volta (1.1 deaths per 1000 admissions) and Upper East (1.8 deaths per 1000 admissions) (S1 Table). Among the cancers that were specified, more deaths were reported from cancer of the liver, breast cancer, and prostate cancer in 2018 (S1 Table). The remaining 13 causes of death are also ranked and presented in Tables 4 and 5.

One of the underlying behavioural causes of death was alcohol-related deaths. Alcohol related deaths placed 17^th^ on the list in 2017 and 2018 (Table 3). Another underlying cause of death of interest was road traffic accidents. Road traffic accidents had a mortality rate of 0.1 deaths in 2018, the highest recorded was in the Upper East Region (0.9 deaths per 1000 admission). Within the category of pregnancy, childbirth and puerperium, maternal hypertensive disorders recorded the most underlying deaths in both 2017 and 2018. This was followed in both years by maternal haemorrhage (Tables 4 and 5), a condition described as excessive bleeding just before or after the delivery of a baby. Additional information is presented in the summary cause of death (S1 and S2 Tables).

## Discussion

This study examined institutional mortality rates and cause of death in Ghana. Between 2014 and 2018, institutional mortality rate reduced nationwide save for four out of ten regions that recorded an increase. The cause of death data showed that chronic non-communicable diseases were the leading cause of death for 2017 and 2018. Certain infections and parasites followed this and respiratory infections, followed by diseases of the digestive system. In similar studies at the University of Ghana hospital in 2018 [22], Korle-Bu teaching hospital [23] and a tertiary Hospital in South-West Nigeria [24], NCDs were the leading causes of death. Furthermore, data from the GHS and the Ghana Statistical Service (GSS) confirm hypertension as a leading NCD in Ghana. According to the GHS, hypertension was placed fourth in 2014 [25] and sixth in 2015 and 2016 [26, 27]. The GSS, GHS, and ICF International [28] reported a hypertension prevalence rate of 13% in 2014.

Our findings support arguments for a double burden of diseases in Ghana [7, 29]. Nearly a decade and a half ago, Adjuik et al. [30] concluded that there were high rates of non-communicable diseases side-by-side infectious disease in South Africa and Bangladesh, but not in East Africa and West Africa. Data from the West Africa sub-region for their paper were sourced from Navrongo in Ghana and two other sub-regions sites. Overall, however, Adjuik et al. [30] found that most causes of death in the four regions (three localities in sub-Saharan Africa combined and Bangladesh) studied were infectious, mirrored mostly by the disproportional prevalence of AIDS and malaria. However, the difference between then and now is that deaths from both malaria and AIDS are decreasing across all these regions [31–34].

The reductions in institutional mortality rate can be explained by the investment in healthcare and the success of national infectious disease control programmes, especially childhood diseases [30]. These healthcare investments in Ghana pertain to health infrastructure, health workforce and capacity building, and expansion of service delivery, especially at the primary healthcare level. Others include health information systems—data collection (improving quality of data) and documentation, supplies, medicines, and logistics.

Additionally, since the onset of the Millennium Development Goals (MDGs), there has been increased efforts to improve healthcare in the worst endowed regions and reduce regional inequities. In 1996, the Community-based Health Planning and Services (CHPS) was piloted in Navrongo. Between 1999 and 2003, CHPS was operationalised in the country and accepted as Ghana’s flagship programme for achieving the Universal Health Coverage (UHC). While it was initially implemented in the Northern Regions of Ghana, it quickly spread to almost every part of the country [35, 36]. Although several studies reveal that the implementation of CHPS is challenged as the national scale-up has deviated from some of the original principles of the Navrongo trial, at the very minimum, CHPS is able to handle common infectious diseases while promoting basic hygiene within most rural contexts [10, 37].

Again, CHPS compounds are an important first point-of-call for promoting maternal and child health and health in general. CHPS compounds are also critical in the healthcare chain for preventing certain harmful health-related practices and ailments. They also serve as an important first point-of-call referral point to receiving healthcare at higher-level healthcare centres. There were 5,868 CHPS compounds spread across the country as of the close of 2018 [38]. This has increased the health workforce significantly in the Northern regions. However, the Upper West and Upper East Regions, two regions with an increase in institutional mortality rate, had the least number of the health workforce in 2016 [11, 27].

Most CHPS compounds are now equipped with at least one midwife who oversees pregnancy and child birth-related issues within the context of rural Ghana [39, 40]. While CHPS may have contributed significantly to the reduction in under-five and maternal mortality, CHPS is inept in other major causal attributions of death, such as NCDs. Even though CHPS provides the widest access to healthcare in the country, the service is not equipped to provide basic NCD support service to common ailments such as hypertension and other heart-related diseases [41]. This deficiency of primary healthcare has serious implications for NCD care in most parts of Ghana, where only CHPS has a reach. NCD care and healthcare investments have been disproportionately concentrated in the city centre to neglect rural areas who also have an equal burden of NCD mortality. These structural deficiencies in the delivery of care in Ghana may partly explain the pattern of NCD mortality. As it stands, the healthcare system is asymmetrical, where it has concentrated most specialised NCD care in tertiary facilities with the least access to the general population.

Nevertheless, we do not call for establishing full-fledged NCD care in rural areas in Ghana due to the small populations, which will attract inadequate patronage for NCD care in these areas, leading to inefficient and wasteful healthcare investment. Intensified education on prevention and early screening for NCDs and information on NCD centres and prompt referral to such centres, accompanied with linking referred patients to such centres, and a feedback loop to the referring centres regarding the patient’s compliance to the referral, will be a more efficient way of investing Ghana’s healthcare resources, with specific reference to NCDs. In terms of the regional variation in our findings, we generally conclude the existence of urban bias in NCD-specific deaths. Greater Accra, the region with the highest NCD deaths, is also the most urbanised region in Ghana [42]. Conversely, the Upper East and Upper West, regions with infections as their first cause-specific death, are mostly rural regions. The Greater Accra Region’s status as the most urbanised and most economically endowed region in Ghana also lends credence to its higher prevalence in hypertension for our study.

In 2014, results from the nationally representative Ghana Demographic and Health Survey [28] revealed that urban residents in Ghana had nearly twice as high hypertension levels compared to rural residents (9.7% for females and 5.6% for males in urban areas; compared to 5% for females and 2.7% for males in rural areas). Urban residents are predisposed to unhealthy food environments, sedentary lifestyle and obesity, which predispose them to hypertension [43–48]. In tandem with this, the increasing proportion of the aged populations in Ghana, particularly in terms of their numbers [42], may also explain the increasing non-communicable diseases our study found [30].

### Limitations

Our data have three key limitations. Firstly, the data were not disaggregated by sex and age. This is problematic because mortality is often both age- and sex-related. Generally, males often have higher death rates than females [2, 49, 50]. The same applies to age categories: deaths are higher among the very young and the very old, than other persons. Data that are not age- and sex-adjusted thus skew true mortality patterns. For instance, age-standardising data helps to better explain the pattern of cause-specific mortality [30]. Another key limitation with this paper is that institution-based data do not reflect the true state of affairs nationally. Particularly, in places like Ghana and sub-Saharan Africa generally, there are problems with access to healthcare facilities [11, 51]. Furthermore, problems with the registration of deaths, and cultural factors that discourage reporting of deaths, although legally required in Ghana, impede capturing the full picture regarding mortality in Ghana. Thus, our findings may not completely be generalisable to Ghana, as institutional data particularly, and morbidity and mortality data in general, are not comprehensive [52].

Secondly, our study found a 2017 total of 14,860 and a 2018 total of 24,714 for the cause of deaths nationally for Ghana, constituting a 66.3% increase over the two years. GHS [38, 53] also reported 13,198 institutional deaths for 2017, and 24,066 for 2018, 82.3% increase over the two years. While the 2018 figures for our data versus those of the GHS are quite close and reflect good data quality for that year, the 2017 data do not. The Institute of Statistical, Social and Economic Research (ISSER) [54] has commented on the need to improve data capture quality for health outcomes in Ghana. Thirdly, regional analyses were adjusted by the regional admissions but not the regional population. This is primarily because people do not necessarily seek healthcare in their regions of residence. Pertaining to deaths particularly, in Ghana, most serious mortality cases seen in healthcare facilities are often referred to the two leading tertiary (also referral) hospitals for management. As articulated above, these referred cases stand the highest chances, all else being equal, of dying in the regions where these two most specialist healthcare facilities are situated [5, 12].

Despite these limitations, institutional mortality data represent an important source of information for death audits and related lessons and healthcare practice improvement and health and healthcare planning and policy formulation. Since the best known available data are reported routinely; yearly, nationally, and by technical personnel, they are a near-excellent source of information that fills the void in mortality-related data available in Ghana. Therefore, irrespective of the above-stated limitations of our data, this paper has critical strengths because it provides a holistic picture of institutionally reported mortality in Ghana; it is based on longitudinal data and applies the most recent WHO’s [16] approved international classification of mortality (ICD-11).

### Conclusions and recommendations

We studied the national mortality pattern of Ghana using institutional data from the Ghana Health Service for 2014 to 2018. We aimed to fill a void in the literature and provide critical information for healthcare and general development planning and policy formulation [30]. We found that over the study period, institutional mortality decreased. This pattern of mortality may result from the effectiveness of the investment in healthcare and the greater efforts to address the inequities in healthcare personnel, particularly in the less endowed regions in Ghana. The study also concludes that over the last two years of the study period (2017 and 2018), hypertensive heart diseases, certain infectious and parasitic diseases such as septicaemia, and respiratory diseases such as pneumonia have taken centre-stage as key underlying causes of mortality in Ghana. NCDs were the leading cause of death in the more urbanised regions while infections were the leading cause of death in the rural regions.

The public health implications of our study is the need to strengthen the health behaviour change communication to the Ghanaian public on alternative nutrition and lifestyle, such as eating healthier, exercising more, and avoiding obesity. Efforts to bridge the hitherto yawning gap between resources, particularly healthcare resource endowments between the northern and southern parts of Ghana, should be boosted. There is also the need to task shift as suggested by other authors [41, 47, 55, 56]; to enhance NCDs care at health facilities. Further research using longitudinal data over a longer period than ours is needed to elucidate the pattern of the cause-specific fully and underlying mortality in Ghana, in both institutionally and non-institutionally reported data.

## Supporting information

S1 Table Underlying cause of death in 2017 by region. (DOCX)

S2 Table Underlying cause of death in 2018 by region. (DOCX)

S1 Data Inst mortlity&cause of death—Ghana—2014-2018. (XLSX)

## Abbreviations

AIDS: Acquired immune deficiency syndrome
CHPS: Community-based Health Planning and Services
CHAG: Christian Health Association Ghana (CHAG)
DHIMS2: District Health Information Management System
GHS: Ghana Health Service
GSS: Ghana Statistical Service
HIV: Human immunodeficiency virus
ICD: International Classification for Diseases
ISSER: Institute of Statistical, Social and Economic Research
MDGs: Millennium Development Goals
NCD: Non-communicable disease
NTDs: Neglected tropical diseases
PPMED: Policy, Planning Monitoring and Evaluation Division
SOP: Standard Operating Procedures
UHC: Universal Health Coverage
UNDESAPD: United Nations Department of Economic and Social Affairs, Population Division
