# Supplementary material for: Institutional mortality rate and cause of death at health facilities in Ghana between 2014 and 2018
Source: PLoS One. 2021 Sep 8;16(9):e0256515. doi: 10.1371/journal.pone.0256515 (PMC8425528; doi:10.1371/journal.pone.0256515)
Supplement: S1 Table — (DOCX) [file pone.0256515.s001.docx]

## S1 Table: Underlying cause of death in 2017 by region

| **Disease Group** | **Underlying cause of death** | **Ashanti** | **Bono Ahafo** | **Central** | **Eastern** | **Greater Accra** | **Northern** | **Volta** | **Upper East** | **Upper West** | **Western** | **Ghana** |
| --- | --- | --- | --- | --- | --- | --- | --- | --- | --- | --- | --- | --- |
| Alcohol related deaths | Alcohol use disorders | 0.1 | 0.1 | 0.1 | 0.1 | 0.2 | 0.0 | 0.1 | 0.4 | 0.0 | 0.1 | 0.1 |
|  | Alcohol poisoning | 0.0 | 0.0 | 0.0 | 0.1 | 0.0 | 0.0 | 0.0 | 0.0 | 0.0 | 0.0 | 0.0 |
| Cancers | Benign neoplasms | 0.0 | 0.1 | 0.1 | 0.0 | 0.1 | 0.0 | 0.1 | 0.0 | 0.0 | 0.0 | 0.0 |
|  | Leukaemia | 0.0 | 0.0 | 0.0 | 0.0 | 0.1 | 0.0 | 0.0 | 0.0 | 0.0 | 0.0 | 0.0 |
|  | Malignant melanoma of skin | 0.0 | 0.0 | 0.0 | 0.0 | 0.0 | 0.0 | 0.0 | 0.0 | 0.0 | 0.0 | 0.0 |
|  | Malignant neoplasm of bladder | 0.0 | 0.0 | 0.0 | 0.0 | 0.0 | 0.0 | 0.0 | 0.0 | 0.0 | 0.0 | 0.0 |
|  | Malignant neoplasm of breast | 0.0 | 0.1 | 0.1 | 0.1 | 0.4 | 0.0 | 0.1 | 0.0 | 0.1 | 0.0 | 0.1 |
|  | Malignant neoplasm of colon, rectum and anus | 0.0 | 0.0 | 0.0 | 0.0 | 0.1 | 0.0 | 0.0 | 0.0 | 0.0 | 0.0 | 0.0 |
|  | Malignant neoplasm of larynx | 0.0 | 0.0 | 0.0 | 0.0 | 0.0 | 0.0 | 0.0 | 0.0 | 0.0 | 0.0 | 0.0 |
|  | Malignant neoplasm of lip, oral cavity and pharynx | 0.0 | 0.0 | 0.0 | 0.0 | 0.0 | 0.0 | 0.0 | 0.0 | 0.0 | 0.0 | 0.0 |
|  | Malignant neoplasm of liver and intrahepatic bile ducts | 0.1 | 0.2 | 0.1 | 0.2 | 0.3 | 0.0 | 0.1 | 0.2 | 0.1 | 0.0 | 0.1 |
|  | Malignant neoplasm of meninges, brain and other parts of central nervous system | 0.0 | 0.0 | 0.0 | 0.0 | 0.0 | 0.0 | 0.0 | 0.0 | 0.0 | 0.0 | 0.0 |
|  | Malignant neoplasm of Oesophagus | 0.0 | 0.0 | 0.0 | 0.0 | 0.0 | 0.0 | 0.0 | 0.0 | 0.0 | 0.0 | 0.0 |
|  | Malignant neoplasm of other and unspecified parts of uterus | 0.0 | 0.0 | 0.0 | 0.0 | 0.2 | 0.0 | 0.0 | 0.0 | 0.0 | 0.0 | 0.0 |
|  | Malignant neoplasm of ovary | 0.0 | 0.0 | 0.0 | 0.0 | 0.1 | 0.0 | 0.0 | 0.0 | 0.0 | 0.0 | 0.0 |
|  | Malignant neoplasm of pancreas | 0.0 | 0.0 | 0.0 | 0.0 | 0.2 | 0.0 | 0.0 | 0.0 | 0.0 | 0.0 | 0.0 |
|  | Malignant neoplasm of prostate | 0.0 | 0.1 | 0.0 | 0.1 | 0.3 | 0.0 | 0.1 | 0.0 | 0.0 | 0.1 | 0.1 |
|  | Malignant neoplasm of stomach | 0.0 | 0.0 | 0.0 | 0.0 | 0.1 | 0.0 | 0.0 | 0.0 | 0.0 | 0.0 | 0.0 |
|  | Malignant neoplasm of trachea, bronchus and lung | 0.0 | 0.0 | 0.0 | 0.0 | 0.2 | 0.0 | 0.0 | 0.0 | 0.0 | 0.0 | 0.0 |
|  | Multiple myeloma and malignant plasma cell neoplasms | 0.0 | 0.0 | 0.0 | 0.0 | 0.0 | 0.0 | 0.0 | 0.0 | 0.0 | 0.0 | 0.0 |
|  | Non-Hodgkin lymphoma | 0.0 | 0.0 | 0.0 | 0.0 | 0.1 | 0.0 | 0.0 | 0.0 | 0.0 | 0.0 | 0.0 |
|  | Other and unspecified malignant neoplasms | 0.0 | 0.2 | 0.1 | 0.1 | 0.7 | 0.0 | 0.2 | 0.0 | 0.1 | 0.1 | 0.1 |
|  | Other diseases of the blood and blood-forming organs and certain disorders involving the immune mechanism | 0.0 | 0.1 | 0.0 | 0.0 | 0.1 | 0.1 | 0.1 | 0.1 | 0.1 | 0.0 | 0.1 |
| Certain infections and parasites | Acute poliomyelitis | 0.0 | 0.0 | 0.0 | 0.0 | 0.0 | 0.0 | 0.0 | 0.0 | 0.0 | 0.0 | 0.0 |
|  | Cholera | 0.0 | 0.0 | 0.0 | 0.0 | 0.0 | 0.0 | 0.0 | 0.0 | 0.0 | 0.0 | 0.0 |
|  | Dengue | 0.0 | 0.0 | 0.0 | 0.0 | 0.0 | 0.0 | 0.0 | 0.0 | 0.0 | 0.0 | 0.0 |
|  | Diphtheria | 0.0 | 0.0 | 0.0 | 0.0 | 0.0 | 0.0 | 0.0 | 0.0 | 0.0 | 0.0 | 0.0 |
|  | Hepatitis B | 0.0 | 0.0 | 0.0 | 0.0 | 0.0 | 0.0 | 0.0 | 0.0 | 0.0 | 0.0 | 0.0 |
|  | Leprosy | 0.0 | 0.0 | 0.0 | 0.0 | 0.0 | 0.0 | 0.0 | 0.0 | 0.0 | 0.0 | 0.0 |
|  | Measles | 0.0 | 0.0 | 0.0 | 0.0 | 0.0 | 0.0 | 0.0 | 0.0 | 0.0 | 0.0 | 0.0 |
|  | Meningococcal infection | 0.0 | 0.0 | 0.0 | 0.0 | 0.0 | 0.0 | 0.0 | 0.0 | 0.0 | 0.0 | 0.0 |
|  | Other and unspecified infectious diseases | 0.4 | 1.0 | 0.3 | 1.4 | 1.3 | 0.1 | 0.7 | 0.4 | 0.3 | 0.5 | 0.6 |
|  | Other tuberculosis | 0.0 | 0.1 | 0.0 | 0.1 | 0.1 | 0.0 | 0.0 | 0.0 | 0.0 | 0.0 | 0.0 |
|  | Other viral haemorrhagic fevers | 0.0 | 0.0 | 0.0 | 0.0 | 0.0 | 0.0 | 0.0 | 0.0 | 0.0 | 0.0 | 0.0 |
|  | Other viral hepatitis | 0.0 | 0.3 | 0.1 | 0.1 | 0.2 | 0.1 | 0.0 | 0.2 | 0.3 | 0.1 | 0.1 |
|  | Plague | 0.0 | 0.0 | 0.0 | 0.0 | 0.0 | 0.0 | 0.0 | 0.0 | 0.0 | 0.0 | 0.0 |
|  | Rabies | 0.0 | 0.0 | 0.0 | 0.0 | 0.0 | 0.0 | 0.0 | 0.0 | 0.0 | 0.0 | 0.0 |
|  | Septicaemia | 0.7 | 1.6 | 1.5 | 1.2 | 1.7 | 0.6 | 1.5 | 2.1 | 0.8 | 1.0 | 1.2 |
|  | Tetanus | 0.0 | 0.0 | 0.0 | 0.0 | 0.0 | 0.0 | 0.1 | 0.0 | 0.0 | 0.0 | 0.0 |
|  | Tuberculosis | 0.0 | 0.0 | 0.0 | 0.0 | 0.0 | 0.0 | 0.0 | 0.0 | 0.0 | 0.0 | 0.0 |
|  | Tuberculosis, confirmed bacteriologically or histologically | 0.1 | 0.3 | 0.2 | 0.4 | 0.2 | 0.0 | 0.2 | 0.4 | 0.3 | 0.1 | 0.2 |
|  | Typhoid and paratyphoid | 0.0 | 0.0 | 0.0 | 0.0 | 0.0 | 0.1 | 0.1 | 0.2 | 0.0 | 0.0 | 0.0 |
|  | Unspecified viral hepatitis | 0.0 | 0.0 | 0.0 | 0.0 | 0.0 | 0.0 | 0.0 | 0.0 | 0.1 | 0.0 | 0.0 |
|  | Viral hepatitis | 0.0 | 0.0 | 0.0 | 0.0 | 0.0 | 0.0 | 0.0 | 0.0 | 0.0 | 0.0 | 0.0 |
|  | Whooping cough | 0.0 | 0.0 | 0.0 | 0.0 | 0.0 | 0.0 | 0.0 | 0.0 | 0.0 | 0.0 | 0.0 |
|  | Yellow fever | 0.0 | 0.0 | 0.0 | 0.0 | 0.0 | 0.0 | 0.0 | 0.0 | 0.0 | 0.0 | 0.0 |
| Chronic non communicable diseases | Diabetes mellitus | 0.3 | 0.6 | 0.5 | 0.8 | 1.3 | 0.1 | 0.3 | 0.6 | 0.1 | 0.6 | 0.5 |
|  | Hypertensive heart diseases | 0.6 | 1.6 | 1.5 | 2.5 | 4.8 | 0.3 | 1.5 | 1.4 | 0.6 | 1.7 | 1.5 |
|  | Ischaemic heart diseases | 0.1 | 0.2 | 0.2 | 0.2 | 0.4 | 0.0 | 0.2 | 0.1 | 0.1 | 0.1 | 0.1 |
|  | Other heart diseases | 0.6 | 1.0 | 0.7 | 1.5 | 2.1 | 0.2 | 1.4 | 1.0 | 0.2 | 0.3 | 0.9 |
| Diseases of the circulatory system | Acute rheumatic fever and chronic rheumatic heart diseases | 0.0 | 0.0 | 0.0 | 0.0 | 0.0 | 0.0 | 0.0 | 0.0 | 0.0 | 0.0 | 0.0 |
|  | Cerebrovascular diseases | 0.4 | 1.2 | 1.0 | 2.5 | 2.7 | 0.2 | 2.3 | 1.1 | 0.5 | 0.6 | 1.2 |
|  | Other and unspecified diseases of the circulatory system | 0.0 | 0.1 | 0.1 | 0.2 | 0.2 | 0.0 | 0.2 | 0.3 | 0.0 | 0.1 | 0.1 |
| Diseases of the digestive system | Appendicitis | 0.0 | 0.0 | 0.0 | 0.0 | 0.0 | 0.0 | 0.0 | 0.0 | 0.0 | 0.0 | 0.0 |
|  | Gastric and duodenal ulcer | 0.1 | 0.1 | 0.0 | 0.1 | 0.1 | 0.0 | 0.2 | 0.1 | 0.1 | 0.0 | 0.1 |
|  | Liver cirrhosis | 0.1 | 0.1 | 0.1 | 0.1 | 0.1 | 0.0 | 0.0 | 0.3 | 0.0 | 0.0 | 0.1 |
|  | Other and unspecified diarrhoeal diseases | 0.1 | 0.2 | 0.2 | 0.1 | 0.2 | 0.2 | 0.1 | 0.3 | 0.1 | 0.1 | 0.2 |
|  | Other diseases of the digestive system | 0.4 | 1.5 | 0.7 | 1.2 | 1.6 | 0.3 | 1.1 | 2.4 | 1.2 | 0.7 | 1.0 |
| Diseases of the skin | Diseases of the skin and subcutaneous tissue | 0.0 | 0.1 | 0.3 | 0.1 | 0.3 | 0.0 | 0.2 | 0.3 | 0.1 | 0.1 | 0.1 |
| Diseases of the nervous system | Other diseases of the nervous system | 0.0 | 0.2 | 0.2 | 0.2 | 0.5 | 0.0 | 0.3 | 0.1 | 0.1 | 0.2 | 0.2 |
| Diseases of the visual system | Diseases of the eye and adnexa | 0.0 | 0.0 | 0.0 | 0.0 | 0.0 | 0.0 | 0.0 | 0.0 | 0.0 | 0.0 | 0.0 |
| Diseases of the ear and mastoid process | Diseases of the ear and mastoid process | 0.0 | 0.0 | 0.0 | 0.0 | 0.0 | 0.0 | 0.0 | 0.0 | 0.0 | 0.0 | 0.0 |
| Diseases of the musculoskeletal system and connective tissue | Diseases of the musculoskeletal system and connective tissue | 0.0 | 0.0 | 0.0 | 0.0 | 0.1 | 0.0 | 0.0 | 0.1 | 0.0 | 0.0 | 0.0 |
| Diseases of the genitourinary system | Glomerular and renal tubulo-interstitial diseases | 0.0 | 0.3 | 0.3 | 0.5 | 0.5 | 0.0 | 0.1 | 0.3 | 0.1 | 0.1 | 0.2 |
|  | Malignant neoplasm of cervix uteri | 0.0 | 0.0 | 0.0 | 0.1 | 0.2 | 0.0 | 0.0 | 0.0 | 0.0 | 0.0 | 0.0 |
|  | Other and unspecified diseases of the genitourinary system | 0.1 | 0.2 | 0.3 | 0.2 | 0.8 | 0.0 | 0.3 | 0.3 | 0.2 | 0.1 | 0.2 |
| External causes of death | Accidental drowning and submersion | 0.0 | 0.0 | 0.0 | 0.0 | 0.0 | 0.0 | 0.0 | 0.0 | 0.0 | 0.0 | 0.0 |
|  | Accidental poisoning by and exposure to noxious substances | 0.0 | 0.0 | 0.0 | 0.0 | 0.0 | 0.0 | 0.0 | 0.0 | 0.0 | 0.0 | 0.0 |
|  | Assault | 0.0 | 0.0 | 0.0 | 0.0 | 0.0 | 0.0 | 0.0 | 0.1 | 0.0 | 0.0 | 0.0 |
|  | Conflict and war | 0.0 | 0.0 | 0.0 | 0.0 | 0.0 | 0.0 | 0.0 | 0.0 | 0.0 | 0.0 | 0.0 |
|  | Drug poisoning | 0.0 | 0.0 | 0.0 | 0.0 | 0.0 | 0.0 | 0.0 | 0.0 | 0.0 | 0.0 | 0.0 |
|  | Exposure to forces of nature | 0.0 | 0.0 | 0.0 | 0.0 | 0.0 | 0.0 | 0.0 | 0.0 | 0.0 | 0.0 | 0.0 |
|  | Exposure to smoke, fire and flames | 0.0 | 0.0 | 0.0 | 0.0 | 0.0 | 0.0 | 0.0 | 0.0 | 0.0 | 0.0 | 0.0 |
|  | Falls | 0.0 | 0.0 | 0.0 | 0.0 | 0.0 | 0.0 | 0.0 | 0.0 | 0.0 | 0.0 | 0.0 |
|  | Food poisoning | 0.0 | 0.0 | 0.0 | 0.0 | 0.0 | 0.0 | 0.0 | 0.0 | 0.0 | 0.0 | 0.0 |
|  | Intentional self-harm | 0.0 | 0.0 | 0.0 | 0.0 | 0.0 | 0.0 | 0.0 | 0.0 | 0.0 | 0.0 | 0.0 |
|  | Other and unspecified external causes | 0.1 | 0.5 | 0.2 | 0.3 | 0.3 | 0.2 | 0.4 | 0.7 | 0.6 | 0.2 | 0.3 |
|  | Other and unspecified poisoning | 0.0 | 0.0 | 0.0 | 0.0 | 0.0 | 0.0 | 0.0 | 0.0 | 0.0 | 0.0 | 0.0 |
| Foetal deaths | Birth trauma | 0.0 | 0.0 | 0.0 | 0.0 | 0.0 | 0.0 | 0.0 | 0.0 | 0.0 | 0.0 | 0.0 |
|  | Congenital hydrocephalus and spine bifida | 0.0 | 0.0 | 0.0 | 0.0 | 0.0 | 0.0 | 0.0 | 0.0 | 0.0 | 0.0 | 0.0 |
|  | Congenital malformations of the heart | 0.0 | 0.0 | 0.0 | 0.0 | 0.2 | 0.0 | 0.0 | 0.0 | 0.0 | 0.0 | 0.0 |
|  | Disorders relating to length of gestation and foetal growth | 0.0 | 0.0 | 0.0 | 0.0 | 0.0 | 0.0 | 0.0 | 0.0 | 0.0 | 0.0 | 0.0 |
|  | Down syndrome and other chromosomal abnormalities | 0.0 | 0.0 | 0.0 | 0.0 | 0.0 | 0.0 | 0.0 | 0.0 | 0.0 | 0.0 | 0.0 |
|  | Foetus and new-born affected by maternal factors and by complications of pregnancy, labour and delivery | 0.0 | 0.0 | 0.0 | 0.0 | 0.0 | 0.0 | 0.0 | 0.0 | 0.0 | 0.0 | 0.0 |
|  | Indirect obstetric deaths | 0.0 | 0.0 | 0.0 | 0.0 | 0.0 | 0.0 | 0.0 | 0.0 | 0.0 | 0.0 | 0.0 |
|  | Intrauterine hypoxia and birth asphyxia | 0.1 | 0.4 | 0.1 | 0.3 | 0.5 | 0.2 | 0.3 | 1.0 | 0.1 | 0.2 | 0.3 |
|  | Low birth weight | 0.0 | 0.0 | 0.0 | 0.0 | 0.0 | 0.0 | 0.0 | 0.1 | 0.0 | 0.0 | 0.0 |
|  | Other and unspecified congenital malformations | 0.0 | 0.0 | 0.0 | 0.0 | 0.1 | 0.0 | 0.0 | 0.0 | 0.0 | 0.0 | 0.0 |
|  | Other and unspecified disorders relating to length of gestation and foetal growth | 0.0 | 0.0 | 0.0 | 0.0 | 0.0 | 0.0 | 0.0 | 0.0 | 0.0 | 0.0 | 0.0 |
|  | Other and unspecified perinatal conditions | 0.1 | 0.2 | 0.2 | 0.3 | 1.1 | 0.0 | 0.2 | 0.1 | 0.2 | 0.2 | 0.2 |
|  | Other direct obstetric deaths | 0.0 | 0.0 | 0.0 | 0.1 | 0.1 | 0.0 | 0.0 | 0.0 | 0.1 | 0.0 | 0.0 |
|  | Prematurity | 0.1 | 0.3 | 0.0 | 0.1 | 0.3 | 0.1 | 0.2 | 0.4 | 0.2 | 0.1 | 0.2 |
|  | Sepsis and other infectious conditions of the new-born | 0.0 | 0.1 | 0.1 | 0.1 | 0.2 | 0.2 | 0.1 | 0.6 | 0.1 | 0.1 | 0.1 |
| HIV | HIV disease with tuberculosis | 0.0 | 0.0 | 0.0 | 0.0 | 0.0 | 0.0 | 0.0 | 0.0 | 0.0 | 0.0 | 0.0 |
|  | Human immunodeficiency virus [HIV] disease | 0.0 | 0.0 | 0.0 | 0.0 | 0.0 | 0.0 | 0.0 | 0.0 | 0.0 | 0.0 | 0.0 |
|  | Other and unspecified HIV disease | 0.3 | 0.6 | 0.3 | 0.4 | 0.5 | 0.0 | 0.5 | 0.3 | 0.4 | 0.2 | 0.3 |
| Malaria | Malaria | 0.0 | 0.0 | 0.0 | 0.0 | 0.0 | 0.0 | 0.0 | 0.0 | 0.0 | 0.0 | 0.0 |
|  | Malaria, parasitological confirmed | 0.1 | 0.2 | 0.1 | 0.1 | 0.1 | 0.4 | 0.2 | 0.3 | 0.1 | 0.1 | 0.2 |
|  | Other and unspecified malaria | 0.1 | 0.2 | 0.1 | 0.1 | 0.0 | 0.3 | 0.1 | 0.5 | 0.1 | 0.1 | 0.1 |
| Malnutrition | Anaemias | 0.3 | 0.7 | 0.7 | 0.9 | 0.8 | 0.5 | 0.9 | 1.8 | 0.6 | 1.1 | 0.7 |
|  | Other and unspecified endocrine, nutritional and metabolic diseases | 0.2 | 0.4 | 0.3 | 0.3 | 0.2 | 0.1 | 0.3 | 0.3 | 0.2 | 0.2 | 0.2 |
|  | Protein-energy malnutrition | 0.0 | 0.1 | 0.1 | 0.1 | 0.2 | 0.2 | 0.0 | 0.2 | 0.0 | 0.0 | 0.1 |
| Mental, behavioural and neurodevelopmental | Alzheimer's disease, dementias | 0.0 | 0.0 | 0.0 | 0.0 | 0.0 | 0.0 | 0.0 | 0.0 | 0.0 | 0.0 | 0.0 |
|  | Drug use disorders | 0.0 | 0.0 | 0.0 | 0.0 | 0.0 | 0.0 | 0.0 | 0.0 | 0.0 | 0.0 | 0.0 |
|  | Other mental and behavioural disorders | 0.0 | 0.0 | 0.0 | 0.0 | 0.1 | 0.0 | 0.0 | 0.0 | 0.0 | 0.0 | 0.0 |
| Neglected tropical diseases | Leishmaniasis | 0.0 | 0.0 | 0.0 | 0.0 | 0.0 | 0.0 | 0.0 | 0.0 | 0.0 | 0.0 | 0.0 |
|  | Meningitis | 0.0 | 0.2 | 0.1 | 0.2 | 0.1 | 0.1 | 0.1 | 0.3 | 0.2 | 0.0 | 0.1 |
|  | Schistosomiasis | 0.0 | 0.0 | 0.0 | 0.0 | 0.0 | 0.0 | 0.0 | 0.0 | 0.0 | 0.0 | 0.0 |
|  | Trypanosomiasis | 0.0 | 0.0 | 0.0 | 0.0 | 0.0 | 0.0 | 0.0 | 0.0 | 0.0 | 0.0 | 0.0 |
| Pregnancy, childbirth and the puerperium | Maternal haemorrhage | 0.0 | 0.0 | 0.0 | 0.1 | 0.0 | 0.0 | 0.0 | 0.0 | 0.0 | 0.0 | 0.0 |
|  | Maternal hypertensive disorders | 0.0 | 0.0 | 0.0 | 0.1 | 0.0 | 0.0 | 0.0 | 0.0 | 0.0 | 0.0 | 0.0 |
|  | Maternal sepsis | 0.0 | 0.0 | 0.0 | 0.0 | 0.0 | 0.0 | 0.0 | 0.0 | 0.0 | 0.0 | 0.0 |
|  | Obstructed labour | 0.0 | 0.0 | 0.0 | 0.0 | 0.0 | 0.0 | 0.0 | 0.0 | 0.0 | 0.0 | 0.0 |
|  | Pregnancy with abortive outcome | 0.0 | 0.0 | 0.0 | 0.0 | 0.0 | 0.0 | 0.0 | 0.0 | 0.0 | 0.0 | 0.0 |
| Respiratory infections | Chronic lower respiratory diseases | 0.0 | 0.1 | 0.1 | 0.0 | 0.1 | 0.1 | 0.1 | 0.2 | 0.1 | 0.0 | 0.1 |
|  | Influenza | 0.0 | 0.0 | 0.0 | 0.0 | 0.0 | 0.0 | 0.0 | 0.0 | 0.0 | 0.0 | 0.0 |
|  | Other acute lower respiratory infections | 0.0 | 0.0 | 0.0 | 0.0 | 0.0 | 0.0 | 0.0 | 0.0 | 0.0 | 0.0 | 0.0 |
|  | Other and unspecified diseases of the respiratory system | 0.6 | 1.5 | 1.0 | 0.9 | 2.0 | 0.2 | 1.5 | 0.7 | 0.3 | 0.4 | 0.9 |
|  | Pneumonia | 0.4 | 1.3 | 0.8 | 1.4 | 1.4 | 0.7 | 1.3 | 2.6 | 0.8 | 0.9 | 1.0 |
| Road accidents | Other transport accidents | 0.0 | 0.0 | 0.0 | 0.0 | 0.0 | 0.0 | 0.0 | 0.0 | 0.0 | 0.0 | 0.0 |
|  | Road traffic accidents | 0.0 | 0.1 | 0.1 | 0.1 | 0.1 | 0.0 | 0.2 | 0.9 | 0.1 | 0.1 | 0.1 |
| Sexually transmitted infections | Infections with a predominantly sexual mode of transmission | 0.0 | 0.0 | 0.0 | 0.0 | 0.0 | 0.0 | 0.0 | 0.0 | 0.0 | 0.0 | 0.0 |
|  | Other and unspecified infections with a predominantly sexual mode of transmission | 0.0 | 0.0 | 0.0 | 0.0 | 0.0 | 0.0 | 0.0 | 0.0 | 0.0 | 0.0 | 0.0 |
|  | Syphilis | 0.0 | 0.0 | 0.0 | 0.0 | 0.0 | 0.0 | 0.0 | 0.0 | 0.0 | 0.0 | 0.0 |
| Symptoms, signs and abnormal clinical and laboratory findings, not elsewhere classified | Symptoms, signs and abnormal clinical and laboratory findings, not elsewhere classified | 0.1 | 0.5 | 0.4 | 0.4 | 0.7 | 0.2 | 0.9 | 0.9 | 0.3 | 0.3 | 0.4 |
| Unspecified | N/A | 0.0 | 0.0 | 0.0 | 0.0 | 0.0 | 0.0 | 0.0 | 0.0 | 0.0 | 0.0 | 0.0 |
